# Optimal sparse coding of song in a size-constrained auditory system?

**DOI:** 10.1186/1471-2202-12-S1-O12

**Published:** 2011-07-18

**Authors:** Jan Clemens, Susanne Schreiber, Olaf Kutzki, Bernhard Ronacher, Sandra Wohlgemuth

**Affiliations:** 1 Department of Biology, Humboldt-Universität zu Berlin, 10115, Germany; 2 Bernstein Center for Computational Neuroscience, Berlin, 10115, Germany; 3 Institute for Theoretical Biology, Humboldt-Universität zu Berlin, 10115, Germany

## 

Optimal coding theory has been successfully applied to understand the principles of organization in many sensory systems. However, these systems were usually large and relatively generic stimulus encoders, like the early visual and auditory system of vertebrates or the early olfactory system in insects [1-3]. Do the principles of optimal coding also apply to small sensory systems with a specialized task and a restricted set of relevant stimuli? We studied the early auditory system of grasshoppers as an example for such a sensory system. These insects use genetically fixed songs to recognize mates with high fidelity. First steps of the processing of song take place in a 3-layer feed-forward network consisting of only a few dozen neurons.

Analyzing the transformation of the neural code for song in grasshoppers, we find that a temporally sparse representation of song is created. Additionally, responses of populations of cells in each of the three network layers get more diverse due to a higher diversity in the stimulus selectivity. That is, while neurons in the first two layers are selective for very similar temporal features of the song, each neuron in the network’s output stage responds to a specific temporal feature.

This transformation has implications for the population code in the network: By asking whether a population decoder needs to incorporate information about which neuron fired which spike to discriminate stimuli optimally, we find that neuronal identity becomes more and more important for an effective read out of the population the higher one ascen^gds in the network [4]. Thereby, an explicit, labeled-line like population code for temporal features of the song is created: This means that each neuron in the output layer signals the presence or absence of a specific temporal feature by the presence or absence of spikes in its response. In contrart, preceding layers encode temporal features implicitly by the temporal patterns of spikes.

Although the creation of a sparse, labeled-line like code resembles the transformations happening in large sensory systems, the small size and restricted task of the early auditory system of grasshoppers leads us to a different conclusion about the objective of these transformations. Early sensory areas in mammals like V1 encode the stimulus largely comprehensively – they filter the stimulus only little according to behavioral relevance as these networks serve as hubs which distribute information to more specific processing stages. In contrast, the grasshoppers’ songs have a genetically fixed structure, freeing the animals from the task to learn the significance of a stimulus feature during life time. This allows grasshoppers to hard-wire and specialize their representation early in the sensory pathway, leading to a compression of the stimulus representation based on behavioral relevance. We have evidence that some of the neurons at the output of the network indeed do encode stimulus features directly related to behavior. Despite being at the very beginning in the grasshopper’s auditory pathway, this representation might thus be similar to higher order areas in vertebrates, as it produces specific representations of behaviorally relevant features.
